# Food security is not the only solution to prevent under-nutrition among 6–59 months old children in Western Amhara region, Ethiopia

**DOI:** 10.1186/s12887-018-1386-2

**Published:** 2019-01-07

**Authors:** Yeshalem Mulugeta Demilew, Abiot Tefera Alem

**Affiliations:** 1 School of Public Health, College of Medicine and Health Sciences, Bahir Dar University, P.O.Box 79, Bahir Dar, Ethiopia; 2 School of Medicine, College of Medicine and Health Sciences, Bahir Dar University, P.O.Box 79, Bahir Dar, Ethiopia

**Keywords:** Stunting, Underweight, Wasting, Food secured and 6–59 months old children

## Abstract

**Background:**

In spite of surplus food production, in Amhara region, a significant number of children had under-nutrition. Investigating factors associated with under-nutrition in food secured households is crucial to design preventive measures. Therefore, the objective of this study was to assess under-nutrition and associated factors among 6–59 months old children in food secured households in Western Amhara Region, Ethiopia.

**Methods:**

A community-based cross-sectional study was performed using interviewer-administered questionnaire on 6–59 months old children from Jun 01–30/ 2017. A multi-stage sampling strategy was used to select study participants. Prevalence of stunting, underweight, wasting and overweight/obesity were computed. Predictors were assessed using logistic regression analysis.

**Result:**

The prevalence of stunting, underweight, wasting and overweight/obesity were 40%, 19.8%, 11.6%, and 2.7%, respectively. Having mother who have no formal education (AOR] =2.21, 95% CI: [1.5, 3.2]), taking less diversified food (AOR =1.7, 95% CI: [1.1, 2.5]), having mother who did not wash her hands before food preparation (AOR =1.46, 95% CI: [1.1, 2.0]) and living in the households where solid wastes managed by scattering in the field (AOR =1.6, 95% CI: [1.1, 2.1]) were predictors of stunting. Whereas, wasting was associated with having illness in the prior two weeks of data collection day (AOR =2.7, 95% CI: [1.6, 4.7]), lack of getting antenatal care (AOR =2.0, 95% CI: [1.1, 3.4]) and taking food less than four times per day (AOR =2.00, 95% CI: [1.2, 3.2]).

**Conclusion:**

The prevalence of under-nutrition was very high. Therefore, health professionals and health extension workers should give nutrition counseling about the frequency and diversity of meal, environmental and personal hygiene by giving emphasis to mothers who have no formal education.

**Electronic supplementary material:**

The online version of this article (10.1186/s12887-018-1386-2) contains supplementary material, which is available to authorized users.

## Background

The nutritional status of children determines their growth, development, health, and survival [1]. Malnutrition is the major risk factor that contributes to morbidity and mortality during the childhood period. Under-nutrition contributes 3.1 million (45%) deaths in under-five years old children [2, 3]. Undernourishment affects both mental and physical growth of survivors which in turn significantly affect their performance and economic growth [4]. Moreover, it leads to central obesity, type 2 diabetes mellitus, cardiovascular disease and hyperlipidemia in later life [5].

Under-nutrition includes stunting, underweight, wasting, and deficiencies of essential vitamins and minerals [3]. Stunting refers to chronic nutrition deficiency which restricts the potential growth of a child [6] whereas wasting indicates acute energy deficiency [3, 7]. Under-nutrition occurs as a result of inadequate intakes of energy and nutrients, such as good quality protein, vitamins and minerals which leads failure to meet body need of nutrients to ensure growth, maintenance, and specific functions [8].

Despite significant effort to eradicate malnutrition in its all forms, the world has seen slow progress in reducing under-nutrition [1]. According to United Nations International Children’s Emergency Fund, World Health Organization and World Bank joint estimate of child malnutrition from 1990 to 2017, the level of stunting reduced from 253.4 million (39.3%) to 150.8 million (22.2%) whereas overweight/obesity increased from 32 million (5%) to 38.3 million (5.6%). In the same report in 2017, wasting affects 50.5million (7.5%) under five years old children in the world [1].

Majority of malnourished children reside in African and Asian countries [2]. In Asia, in 2017, the prevalence of stunting, wasting and overweight in under 5 years old children was 55%, 69% and 46%, respectively. Similarly, in 2017, 39%, 27% and 25% of under 5 years old African children were stunted, wasted and overweight, respectively. According to 2016 Ethiopian Demographic and Health Survey report, the prevalence of stunting, underweight and wasting were 38%, 24% and 10%, respectively [9].

The prevalence of under-nutrition among children in food secured households was not significantly different from the magnitude of the problem in children who reside in food insecure households. For example, in food secure households of Nepal, the prevalence of stunting, underweight, and wasting were 34.2%, 19.3% and 7%, respectively. Whereas, in food insecure households of Nepal, 44.7%, 26.4%, and 10.2% of under 5 years old children were stunted, underweight and wasted, respectively [10]. The same is true in Ethiopian context, in Saesie Tsaeda-Emba District, the prevalence of under-nutrition has no significant difference in food secure and food insecure households (52.1%Vs 46.1%) [11].

The causes of under-nutrition are grouped under three broad classifications such as immediate, underlying and basic causes. Immediate causes are mostly related to poor diet or severe and repeated infections, particularly in underprivileged populations. Immediate causes, in turn, are affected by a general standard of living, the environmental conditions, and whether a population is able to meet its basic needs such as food, housing, and healthcare. Many studies showed the association of mothers’ hand washing practice and the risk of having under-nutrition [12]. Having antenatal care (ANC) visit significantly associated with child malnutrition. According to a study done in Nigeria, children whose mothers had low ANC visits during pregnancy were more likely to be malnourished [13]. Further, these underlying causes are related to basic causes like ideology, culture, religion, education, resource, political etc. [14, 15].

In the study area, there is a scarcity of information on the prevalence and associated factors of under-nutrition among 6–59 months old children in food secure household. Children in the age of 6–59 months are at high risk of nutritional deficiency. Identifying the contributing factors for under-nutrition among 6–59 months old children in food secured household is important to set sustainable and effective nutritional interventions. Thus, this study was designed to assess the prevalence of under-nutrition and associated factors among 6–59 months old children in food secure household.

## Methods

### Study setting

This study was conducted in Western Amhara Region, Northwestern part of Ethiopia. This part of the region is composed from five zones such as Agew Awi, West Gojjam, East Gojjam, North Gondar and South Gondar Zones. The total population of the study area is 12,575,929 and the number of under-five years old children is 628,796.

### Study design and population

The study utilized cross-sectional study design. All 6–59 months old children who reside in food secure households in the study area were eligible to participate in the study.

### Sample size and sampling procedure

The sample size for this study was determined using single population proportion formula by assuming the proportion of under-five years old children with stunting in food secured households 46% [11], with 95% confidence level and marginal error 5%. The calculated sample was multiplied by design effect 2, since multi-stage sampling technique was used and 10% non-response rate was added. Accordingly, the calculated sample size was 841.

Multi-stage sampling strategy was used to select study participants. First, two zones (East and West Gojjam zones) were selected from five zones in the study area using Simple Random Sampling (SRS) technique. Sample Woredas were also selected from East and West Gojjam zones by SRS technique. Then, sample Kebeles (the smallest administrative unite in Ethiopia) within selected Woredas were chosen by SRS technique, again. Finally, study participants were selected by SRS technique using list of 6–59 months old children registered during food security assessment as a sampling frame. In the households with more than one eligible 6–59 months old children, one child was selected by lottery method.

### Data collection tool and procedures

Data were collected by interviewing the study participants using pretested, structured questionnaire (Additional file 1). The questionnaire consisted of socio demographic and obstetric characteristics, environmental factors, anthropometry, child health and caring practice. The questionnaire was developed in English referring related literature [11, 16, 17]. The questionnaire translated to Amharic (the local language) and back-translated to English by experts of both languages. Eight experienced nurses and three public health professionals were recruited as a data collector and supervisor, respectively. Interviews with mothers were conducted considering privacy at the participant’s home.

### Measurement

Before data collection, food security status of the household was assessed using questionnaires adapted from household food insecurity access scale which was previously validated for use in developing countries [18, 19]. Twenty seven questions were used to assess food security status of the household. A household which had experience of less than the first 2 food insecurity indicators from the 27 were considered as food secured household. But, a household which had experience of more than the first 2 food insecurity indicators from the 27 were considered as food insecure household. Then, 6–59 months old children reside in food secured households were included in this study.

Dietary diversity score was calculated by summing the number of food groups consumed over the 24-h recall period. Children who took four or more food groups were labeled as appropriate dietary diversity score otherwise inappropriate dietary diversity score.

Height/length and weight measurement of children were taken using calibrated equipments and standardized techniques. Functionality of equipments used to measure weight and height/length was checked each day before the actual data collection and each measurement. Weight was measured to the nearest 0.1 kg using an easily portable weighing scale (SECA Germany) for children above 24 months and salter scale for less than 24 months old children. Children were weighed in lightly indoor clothing and barefoot.

Height/length was measured by a vertical or horizontal measuring board. During height measurement, each child stood erect on the measuring board without shoes. During the procedure children’s heels, buttock, shoulder, and back of the head touch the board. During length measurement, each child lied on the measuring board without shoes and by making his body straight and his hands on the side. The measurer pushed the headpiece of the measuring board until it touches the vertex of the head and read at eye level to the nearest 0.1 cm. For all measurements, two readings were taken from each child, and the average was recorded on the questionnaire.

Children’s age, sex, weight, length/height were entered into Emergency Nutrition Assessment (ENA) for SMART 2011 software (SMART Tech, Calgary, AB, USA) to determine the level of stunting (height for age *z*-scores), underweight (weight for age *z*-scores), and wasting (weight for height *z*-score). Accordingly, based on the WHO 2006 reference [20], children who were below − 2 and − 3 SDs for height for age were defined as stunted and severely stunted, respectively. Children who were below − 2 and − 3 SDs for weight for age were considered as underweight and severely underweight, respectively. Children who were below − 2 and − 3 SDs for weight for height were taken as wasted and severely wasted, respectively. When weight for height is above + 2 SDs, it was taken as overweight/obesity.

### Data quality control

Three days training was given for data collectors and supervisors. Pre-test was carried out on eligible children in similar settings not included in the study. The supervisors and investigators performed close supervision during the whole period of data collection. Completed questionnaires were checked up before collecting from data collectors in a daily base. Functionality of weight measuring scale was checked before weighing each child.

### Data processing and analysis

Data entry and analysis was performed using SPSS version 23 software. The ENA for SMART 2011software was used to generate anthropometric measurement indices. Dependant variables were stunting and wasting. Socio-demographic and obstetric characteristics, feeding practice and environmental factors were considered as independent variables. The prevalence of malnutrition was determined. Logistic regression was applied to identify risk factors of under- nutrition. Independent variables with a *p*-value of < 0.2 during the bivariate analysis were taken to the multivariable logistic regression model and *p*-value < 0.05 was taken as statistically significant.

### Ethical consideration

The protocol of this study was approved by Ethical Review Board of Bahir Dar University. Zonal and Woreda Health Bureaus gave letter of permission to do the study. Since the study imposes less than minimal risk, mothers/ care givers gave verbal consent to participate in the study after provision of full information about the risk and benefit of the study. Confidentiality of the study participants was maintained throughout the whole study period. Counseling was given to the mother on child caring and environmental sanitation. Children with nutritional problem were referred to the nearby health institution for management service.

## Result

A total of 841 mother-child pairs were initially enrolled in this study but 815 participants gave complete data, which makes the response rate of 96.9%. The mean (+/- SD) age of children was 29.38 (**±**16.0SD) months. Ninety nine percent of the study participants were Amhara in their Ethnicity. Regarding their religion, almost all (99.4%) respondents were orthodox christens.

Majority (92.2%) of children’s mothers/ caregivers were married. Only 24.3% of mothers and 30% of fathers had formal education. About 78.7% of mothers were housewives and 64.5% of fathers were farmers. About 88.7% of children live with their biological parents. Nearly two in three, 62.3% fathers made decision on use of money in the household (Table 1).

**Table 1:** Socio- demographic characteristics of respondents in food secured households of Western Amhara region, Ethiopia, June 2017, *n* = 815

Variable	Frequency (n)	Percent (%)
Sex		
Male	439	53.9
Female	376	46.1
Age of the child (months)		
6–12	160	19.6
13–24	218	26.8
25–59	437	53.6
Age of the mother (years)		
< 24	115	14.1
25–34	467	57.3
> 35	233	28.6
Religion		
Orthodox	810	99.4
Muslim	5	0.6
Ethnicity		
Amhara	807	99.0
Agew	8	1.0
Educational status of the mother		
Have no formal education	617	75.7
Have formal education	198	24.3
Occupational status of the mother		
Housewife	642	78.7
Merchant	104	12.8
Government employee	69	8.5
Family size		
< 4	383	47.0
> 4	432	53.0
Marital status of the mother		
Married	751	92.2
Divorced/ Single/Widowed	64	7.8
The child live with		
Both biological parents	723	88.7
The mother only	70	8.6
Grand parents	22	2.7
Care givers for the child		
Both parents	433	53.1
The mother only	358	43.9
Grandmother	24	3.0
Decision maker on use of money in the household		
The father only	508	62.3
Both parents	250	30.7
The mother only	57	7.0

### Nutritional status of children

The study revealed that 40% and 13.5% of children were stunted and severely stunted, respectively. Among 19.8% of children who had underweight, 4.8% of them were severely underweight. The prevalence of wasting and severe wasting were 11.6% and 4.2%, respectively. Additionally, 2.7% of children had overweight/obesity (Table 2).

**Table 2:** Nutritional status of 6–59 months old children in food secured households of Western Amhara region, Ethiopia, June 2017 (*N* = 815)

Variable	Frequency (*n* = 815)	Percentage (%)
Under weight	122	15.0
Severely under weight	39	4.8
Normal weight	654	80.2
Stunted	216	26.5
Severely stunted	110	13.5
Not stunted	489	60
Wasted	60	7.4
Severely wasted	34	4.2
Overweight/obesity	22	2.7
Not wasted	699	85.8

### Factors associated with stunting

Factors associated with stunting on bivariate logistic regression analysis were dietary diversity, initiation of complementary feeding, educational status of the mother, possession of television, solid waste management practice, hand washing practice of the mother before food preparation and after cleaning the baby (Table 3).

**Table 3:** Factors associated with stunting of 6–59 months old children in food secured households of Western Amhara region, Ethiopia, June 2017 (*N* = 815)

Variable	Stunted		COR (95% CI)	AOR (95% CI)
	Yes	No		
Dietary diversity				
Appropriate (>4food groups)	33(4.0)	87(10.7)	1.00	1.00
Inappropriate (<4food groups)	293(36.0)	402(49.3)	1.9(1.3,2.9)	1.70(1.1,2.5)
Educational status of the mother				
Have no formal education	88(10.8)	529(64.9)	2.47(1.7,3.5)	2.21(1.5,3.2)
Have formal education	22(2.7)	176(21.6)	1.00	1.00
Initiation of complementary food				
At 6 month	192(23.6)	337(41.3)	1.00	
Before 6 month	49(6.0)	53(6.5)	1.6(1.1,2.6)	
After 6 month	85(10.4)	99 (12.2)	1.5 (1.1,2.1)	
Have Television				
Yes	29 (3.6)	83 (10.2)	1.00	1.00
No	297 (36.4)	406 (49.8)	2.09 (1.3,3.2)	1.71 (1.1,2.6)
The mother wash her hand before food preparation				
Yes	228 (28.0)	385 (47.2)	1.00	1.00
No	98 (12.0)	104 (12.8)	1.59 (1.2,2.1)	1.46 (1.1,2.0)
The mother wash her hand after cleaning the baby				
Yes	240 (29.4)	394 (48.3)	1.00	
No	86 (10.6)	95 (11.7)	1.48 (1.1,2.1)	
Solid waste management				
Burn	112 (13.7)	243 (29.8)	1.00	1.00
Scattered in the field	214 (26.3)	246 (30.2)	1.88 (1.4,2.5)	1.60 (1.1,2.1)

*AOR* Adjusted Odds Ratio, *COR* Crude Odds Ratio, *95%CI* 95 % confidence interval

According to the multiple logistic regression analysis, children whose mothers have no formal education had over twice odds of having stunting compared with children whose mothers have formal education (AOR] =2.21, 95% CI: [1.5, 3.2]). Children who take less than four food groups per day had 1.7 times higher odds to have stunting than children who take four or more food groups (AOR =1.7, 95% CI: [1.1, 2.5]).

Children whose mothers do not wash their hands before food preparation were 1.4 times prone to have stunting than their counterparts (AOR =1.46, 95% CI: [1.1, 2.0]). Children who live in the household have no television had 1.7 times a higher probability to be stunted than their counterparts (AOR =1.71, 95% CI: [1.1, 2.6]). Children who lived in the households where solid wastes managed by scattering in the field had 1.6 times high probability to be stunted compared with children live in the households solid wastes managed by burning it (AOR =1.6, 95% CI: [1.1, 2.1]) (Table 3).

### Factors associated with wasting

Bivariate logistic regression analysis showed that possession of television, type of delivery, sex of the child, ANC visit and illness in the last 2 weeks prior to the date of data collection day were statistically associated with wasting. In the multiple logistic regression analysis, children who had illness in the prior 2 weeks of data collection day had 2.7 times higher odds to have wasting than children who were not ill (AOR =2.7, 95% CI: [1.6, 4.7]).

Children born at home had 2.6 times higher probability to have wasting than children born in the health institution (AOR =2.66, 95% CI: [1.5, 4.6]). Children who live in the household have television had 3.09 times higher risk to be wasted than children who live in the household have television (AOR =3.09, 95% CI: [1.3, 7.4]).

Children whose mothers do not attend ANC during pregnancy had 2 times higher probability to be wasted compared with their counterparts (AOR =2.0, 95% CI: [1.1, 3.4]). Moreover, children who take food less than four times per day had 2 times higher risk to have wasting than children who took four or more meals per day (AOR =2.00, 95% CI: [1.2, 3.2]) (Table 4).

**Table 4:** Factors associated with wasting of 6–59 months old children in food secured households in Western Amhara region, Ethiopia, June 2017 (*N* = 815)

Variable	Wasting		COR (95% CI)	AOR (95% CI)
	Yes	No		
Have Television				
Yes	6 (0.7)	106 (13.0)	1.00	100
No	88(10.8)	615 (75.5)	2.52 (1.1,5.9)	3.09 (1.3,7.4)
Place of delivery				
Institution	18 (2.2)	236 (29.0)	1.00	100
Home	76 (9.3)	485 (59.5)	2.05 (1.2,3.5)	2.66 (1.5,4.6)
Sex of the child				
Male	60 (7.4)	379 (46.5)	1.5 (1.1,2.5)	
Female	34 (4.2)	342 (41.9)	1.00	
Frequency of food intake				
< 3times per day	27 (3.3)	128 (15.7)	1.86 (1.1,3.0)	2.00 (1.2,3.2)
> 3times per day	67 (8.2)	593 (72.8)	1.00	1.00
ANC visit				
Yes	73 (9.0)	623 (76.4)	1.00	1.00
No	21 (2.6)	98 (12.0)	1.82 (1.1,3.1)	2.00 (1.1,3.4)
Illness in the last two weeks				
Yes	24 (2.9)	77 (9.5)	2.86 (1.7,4.8)	2.7 (1.6,4.7)
No	70 (8.6)	644 (79.0)	1.00	1.00

*AOR* Adjusted Odds Ratio, *COR* Crude Odds Ratio, *95% CI* 95 % confidence interval

## Discussion

In this study, 40% (95% CI, 36.0, 43.0) of children were stunted. This indicates the high magnitude of stunting in food secured households which showed that food security is necessary but not the only solution to tackle under-nutrition. This finding is consistent with the national report (38%) [9] and studies done in Shashemene hospital (38.3%) [21], Guto Gida District (41.78%) [22], rural Ethiopia (41.7%) [23] and Indonesia (37%) [24].

On the other hand, this prevalence is lower than the study findings in Ethiopia those reported the prevalence of stunting ranged from 45.8%–57.1% [16, 25, 26], Uganda (51%) [27], Nepal (55.7) [28] and Vietnam (44.3%) [29]. The discrepancy might be due to the difference in the study subjects. This study was conducted among children who lived in the food secured households but the previous studies were done in both food secure and insecure households.

Whereas, this finding is higher than the study findings in Afambo district (32.2%) [30], Kenya (23.3%) [7], Northern Ghana (28.2%) [31], Indonesia (35.1%) [32] and Brazil (9.1%) [33]. The high prevalence of stunting in this study might be due to the socio-demographic and cultural difference among the respondents. In this study, majority of the respondents have no formal education which in turn affects child feeding practice and health-seeking behavior.

The prevalence of underweight was 19.8% (95% CI: 17.1, 22.6). This finding is in line with the study findings in Haramaya district (21%) [25], Uganda (20.7%) [27] and Northern Ghana (19.3%) [31]. On the other hand, it is lower than the study findings in Ethiopia those reported the magnitude of underweight ranging from 23.5%–39.5% [9, 22, 26, 30, 34], Nepal (37%–41.4%) [28, 35] and Vietnam (31.8%). This might also be due to time gap and the difference between the study subjects and child feeding practice. Whereas, it is higher than the study findings in Indonesia (12%) [24] and Brazil (9.8%) [33]. This discrepancy might be due to the difference in the study settings.

In this study, the prevalence of wasting was 11.6% (95% CI: 9.5, 13.7). This finding is in agreement with the study findings in Ethiopia (9.7%–13.4%) [17, 22, 23, 25], Northern Ghana (9.9%) [31] and Indonesia (12%) [24]. On the other hand, it is lower than the study findings in Tahtay Adiyabo Woreda (17.8%) [26], Shashemene hospital (25.2%) [21], Nepal (18,6%) [28] and Vietnam (11.9%). Whereas, it is higher than the study findings in Lalibela (8.9%) [34], Uganda (5.2%) [27] and Brazil (2.6%) [33].

Educational status of the mother was significantly associated with stunting. Children whose mothers have no formal education were more likely to be stunted compared with children whose mothers have formal education. This finding was consistent with previous study findings in Ethiopia [21, 26], Nigeria [36], Iran [37] and Vietnam [29]. This might be due to the fact that educated mothers have a higher probability to expose and understand nutrition messages than non-educated mothers. Besides, educated mothers were more likely to have autonomy, which in turn influences health-related decisions and purchasing food items that improve the child’s access to good quality food.

Children who take less than four food groups per day had a higher probability to have stunting than children who take four or more food groups. This finding is supported by the study findings in Guto Gida district, Ethiopia [22], Ghana [31] and Nepal [38]. The possible explanation to this is that children who take undiversified food were less likely to meet the nutrient requirement which results in failure to thrive.

Hand washing practice of the mother has a positive significant association with stunting. Children whose mothers do not wash their hands before food preparation were at a higher risk to have stunting than their counterparts. This finding is similar to the study finding in Uganda [27]. Hand washing during the critical periods is essential to prevent diarrhea and other infectious diseases among children, which in turn reduce the probability of having stunting.

Children who live in the households where solid wastes managed by scattering in the field had a higher probability to be stunted compared with children who live in the household solid wastes managed by burning. This finding is in agreement with the study finding in Brazil in which poor environmental sanitation was a strong predictor of stunting [33]. This is because solid wastes lying around the household attracts flies, rats, and other creatures that in turn spread infectious disease. Illness affects the nutritional status of children.

Children who were ill in the prior 2 weeks of data collection day were more likely to have wasting than children who were not ill. This finding is consistent with previous study findings in developing countries [16, 25, 26, 36, 39, 40]. This is due to the fact that illness decreases appetite and interfere digestion and absorption of nutrients which directly lead to under-nutrition and by reducing the immune response it exacerbates illness.

Children whose mothers do not attend ANC during pregnancy had a higher probability to be wasted compared with their counterparts. This finding is supported by previous study findings in Ethiopia [25, 30, 41]. The reason for this is mothers who have ANC visit were more likely to get nutrition education which directly affects child feeding practice and health-seeking behavior.

Children who take food less than four times per day were 2 times more likely to develop wasting than their counterparts who took four or more meals per day. This finding is similar to the study finding in Nepal [38]. This is because children who take less than four meals daily were less likely to meet nutrient demand which results in failure to gain weight.

Place of delivery was another predictor for wasting. Children who were born at home had greater probability to be wasted than children who were born at the health institution. This finding is consistent with the study finding in Burundi [42]. Mothers who give birth at home were less likely to get nutrition messages. This directly affects their child feeding practice. Poor feeding practice in turn predispose to under-nutrition.

Children who live in the household have no television were more likely to be stunted and wasted than their counterparts. This finding is in line with the study finding in Ethiopia [43]. Mothers who have television can access information about child feeding practice and health related issues from the media which directly affect feeding practice and health-seeking behavior.

## Conclusion and recommendation

The prevalence of under-nutrition was very high. Taking less diversified meal, scattering solid wastes around the house, having mother that have no formal education and poor hand washing practice of the mother were predictors of stunting. Taking less than four meals per day, giving birth at home, have no television, being ill in the prior 2 weeks of data collection day and whose other have no ANC visits during pregnancy were positively associated with wasting. Therefore, health professionals and health extension workers should give nutrition counseling about the frequency and diversity of diet, environmental and personal hygiene by giving emphasis to mothers who have no formal education.

### Strength of the study

Being a community-based study with a house to house interview make the study representative.

## Limitation of the study

Due to recall bias, initiation of complementary feeding, place of delivery, ANC visit and age of the mother and children may be under or over reported. Another limitation of this study is the absence of data on intestinal parasites.

## Additional file

Additional file 1: Questionaire which was used to collect data for this study. (DOCX 48 kb)

## Abbreviations

ANC: Antenatal care
AOR: Adjusted odd ratio
SD: Standard deviation
SPSS: Statistical package for social science
WHO: World Health Organization

## References

[1] UNICEF / WHO / World Bank Group: Levels and Trends in child Malnutrition, Joint child malnutrition estimates 2018 (UNICEF-WHO-WB). http://datatopics.worldbank.org/child-malnutrition/. 2018.

[2] UNICEF: Improving Child Nutrition:The achievable imperative for global progress. In*.* New York, NY 10017, USA; 2013.

[3] Bhutta ZA, Das JK, Rizvi A, Gaffey MF, Walker N, Horton S, Webb P, Lartey A, Black RE. The Lancet Nutrition Interventions Review Group atMaCNSG: Evidence-based interventions for improvement of maternal and child nutrition: what can be done and at what cost? Lancet. 2013; 10.1016/S0140-6736(1013)60996-60994. 10.1016/S0140-6736(13)60996-4 23746776

[4] Cesar GV, Linda A, Caroline F, Pedro CH, Reynaldo M, Linda R (2008). Maternal and child undernutrition: consequences for adult health and human capital. Lancet.

[5] Martins VJB, Florêncio TMMT, Grillo LP, Franco MCP, Martins PA, Clemente PG, Santos CDL, Vieira MFA, Sawaya AL (2011). Long-Lasting Effects of Undernutrition. Int J Environ Res Public Health.

[6] World Health Organization (2006). WHO child growth standards : length/heightfor-age, weight-for-age, weight-for-length, weight-forheight and body mass index-for-age : methods and development.

[7] Shinsugi C, Matsumura M, Karama M, Tanaka J, Changoma M, Kaneko S (2015). Factors associated with stunting among children according to the level of food insecurity in the household: a cross-sectional study in a rural community of Southeastern Kenya. BMC Public Health.

[8] Rahman AE, Moinuddin M, Molla M, Worku A, Hurt L, Kirkwood B, Mohan SB, Mazumder S, Bhutta Z, Raza F (2014). Childhood diarrhoeal deaths in seven low-and middle-income countries. Bull World Health Organ.

[9] Central Statistical Agency (CSA) [Ethiopia] and ICF (2016). Ethiopia Demographic and Health Survey.

[10] HelenKellerInternational: Household food insecurity and nutritional status of children aged 6–23 months in Kailali district of Nepal. In*.* New work USA; 2010.

[11] Amaha K, Afework M, Omer S (2015). Nutritional status of children (6-59 months) from food secure and food insecure households in rural communities of Saesie Tsaeda-Emba District, Tigray, North Ethiopia: comparative study. Int J Food Sci Nutr.

[12] Rah JH, Cronin AA, Badgaiyan B, Aguayo VM, Coates S, Ahmed S (2015). Household sanitation and personal hygiene practices are associated with child stunting in rural India: a cross-sectional analysis of surveys. BMJ Open.

[13] Hamel C, Enne J, Omer K, Ayara N, Yarima Y, Cockcroft A, Andersson N (2015). Childhood malnutrition is associated with maternal care during pregnancy and childbirth: a cross-sectional study in Bauchi and Cross River States, Nigeria. J Public Health Res.

[14] Pat P, Roy CH: Addressing the Underlying and Basic Causes of Child Undernutrition in Developing Countries:What Works And Why? *Ministry of Foreign Affairs of Denmark* 2009.

[15] Robert EB, Lindsay HA, Zulfiqar AB, Laura EC, Mercedes dO, Majid E, Colin M, Juan R. maternal and child undernutrition: global and regional exposures and health consequences. Lancet. 2008. 10.1016/S0140-6736(07)61690-0.

[16] Demilew YM, Tafere TE, Abitew DB (2017). Infant and young child feeding practice among mothers with 0–24 months old children in Slum areas of Bahir Dar City, Ethiopia. Int Breastfeed J.

[17] Asfaw M, Wondaferash M, Taha M, Dube L (2015). Prevalence of undernutrition and associated factors among children aged between six to fifty nine months in Bule Hora district, South Ethiopia. BMC Public Health.

[18] Coates J, Swindale A, Bilinsky P (2007). Household Food Insecurity Access Scale (HFIAS) for Measurement of Household Food Access: Indicator Guide (v. 3).

[19] Gebreyesus SH, Lunde T, Mariam DH, Woldehanna T, Lindtjørn B. Is the adapted Household Food Insecurity Access Scale (HFIAS) developed internationally to measure food insecurity valid in urban and rural households of Ethiopia? BMC Nutr. 2015;1(2) http://www.biomedcentral.com/bmcnutr/content/1/1/2.

[20] WHO: WHO Child Growth Standards. France; 206.

[21] Zemenu Yohannes Kassa, Tsigereda Behailu, Alemu Mekonnen, Mesfine Teshome, Sintayehu Yeshitila: Malnutrition and associated factors among under five children (6–59 Months) at Shashemene Referral Hospital, West Arsi Zone, Oromia, Ethiopia. Curr Pediatr Res 2017, 21(2).

[22] Adeba A, Garoma S, Fekadu H, Garoma W. Prevalence’s of Wasting and its Associated Factors of Children among 6–59 Months Age in Guto Gida District, Oromia Regional State, Ethiopia. J Food Process Technol. 2014;5(1). 10.4172/2157-7110.1000289.

[23] Endris N, Asefa H, Dube L. Prevalence of malnutrition and associated factors among children in rural Ethiopia. Biomed Res Int. 2017;2017 10.1155/2017/6587853. 10.1155/2017/6587853 PMC5449753 28596966

[24] Blaney S, Februhartanty J, Sukotjo S (2015). Feeding practices among Indonesian children above six months of age: a literature review on their magnitude and quality (part 1). Asia Pac J Clin Nutr.

[25] Yisak H, Gobena T, Mesfin F (2015). Prevalence and risk factors for under nutrition among children under five at Haramaya district, Eastern Ethiopia. BMC Pediatrics.

[26] Tamiru MW, Tolessa BE, Abera SF (2015). Under Nutrition and Associated Factors Among Under-Five Age Children of Kunama Ethnic Groups in Tahtay Adiyabo Woreda, Tigray Regional State, Ethiopia: Community Based Study. Int J Food Sci Nutr.

[27] Bukusuba J, Kaaya AN, Atukwase A (2017). Risk Factors for Stunted Growth among Children Aged 6–59 Months in Rural Uganda. Int J Nutr.

[28] Khatri RB, Mishra SR, Khanal V, Choulagai B: Factors associated with underweight among children of former-Kamaiyas in Nepal. *FrontPublicHealth* 2015, 3(11):doi:10.3389/fpubh.2015.00011. 10.3389/fpubh.2015.00011 PMC4310217 25688344

[29] Hien NN, Kam S (2008). Nutritional status and the characteristics related to malnutrition in children under five years of age in Nghean, Vietnam. J Prev Med Public Health.

[30] Liben ML, Abuhay T, Haile Y (2016). Determinants of child malnutrition among agro pastorals in northeastern Ethiopia: a cross-sectional study. J Health Sci.

[31] Ali Z, Saaka M, Adams A-G, Kamwininaang SK, Abizari A-R. The effect of maternal and child factors on stunting, wasting and underweight among preschool children in northern Ghana. BMC Nutrition. 2017;3. 10.1186/s40795-017-0154-2 PMC7050753 32153813

[32] Torlesse H, Cronin AA, Sebayang SK, Nandy R (2016). Determinants of stunting in Indonesian children: evidence from a cross-sectional survey indicate a prominent role for the water, sanitation and hygiene sector in stunting reduction. BMC Public Health.

[33] Vitolo MR, Gama CM, Bortolini GA, Campagnolo PDB, Drachler ML (2008). Some risk factors associated with overweight, stunting and wasting among children under 5 years old. J Pediatr.

[34] BM Y, Amsalu F, Bikes D (2014). Prevalence and Factors Associated with Stunting, Underweight and Wasting: A Community Based Cross Sectional Study among Children Age 6–59 Months at Lalibela Town, Northern Ethiopia. J Nutr Disorders Ther.

[35] Adhikari D, Khatri RB, Paudel YR, Poudyal AK (2017). Factors Associated with Underweight among Under-Five Children in Eastern Nepal: Community-Based Cross-sectional Study. Front Public Health.

[36] Akombi BJ, Agho KE, Merom D, Hall JJ, Renzah A (2017). Multilevel Analysis of Factors Associated with Wasting and Underweight among Children Under-Five Years in Nigeria. Nutrients.

[37] Mohseni M, Aryankhesal A, Kalantari N (2017). Factors associated with malnutrition among under five-year-old children in Iran: a systematic review. Ann Trop Med Public Health.

[38] Ruwali D (2011). Nutritional status of children under five years of age and factors associated in Padampur VDC, Chitwan. Health Prospect.

[39] Gezahegn Y, Kassahun W, Dube L (2017). Factors associated with acute malnutrition among south Sudanese children in Tierkidi refugee camp: a case-control study. Qual Prim Care.

[40] Yassin MM, Taha MAE, Jamiea SMA (2016). Risk factors associated with wasting among children aged 6 to 24 months old in Gaza strip. Int J Med.

[41] Abera L, Dejene T, Laelago T (2017). Prevalence of malnutrition and associated factors in children aged 6–59 months among rural dwellers of damot gale district, south Ethiopia: community based cross sectional study. Int J Equity Health.

[42] Nkurunziza S, Meessen B, Geertruyden J-PV, Korachais C (2017). Determinants of stunting and severe stunting among Burundian children aged 6–23 months: evidence from a national cross-sectional household survey, 2014. BMC Pediatrics.

[43] Ali AM, Batu MM, Kaushik KK (2016). Socio-economic determinants of nutritional status of children in Ethiopia. Int J Sci Res Publications.

