# Supplementary material for: Food security is not the only solution to prevent under-nutrition among 6–59 months old children in Western Amhara region, Ethiopia
Source: BMC Pediatr. 2019 Jan 7;19:7. doi: 10.1186/s12887-018-1386-2 (PMC6323821; doi:10.1186/s12887-018-1386-2)
Supplement: Supplementary file 1 — Questionaire which was used to collect data for this study. (DOCX 48 kb) [file 12887_2018_1386_MOESM1_ESM.docx]

### Structured English questionnaire

Date of the interview-------------------------------

Interviewer Id No -------------------------------

Time of starting interview--------------------

Time of completing interview-------------------

Name of the cluster ----------------------------

Instruction – indicate the answer by encircle the number which contains the correct answer or write the correct answer in the blank space.

Table 1: Socio-demographic related questions

| **S.No** | **Variable** | **Response** | | | | | | |
| --- | --- | --- | --- | --- | --- | --- | --- | --- |
|  | **Did you have known medical or surgical illness? If no start interview** | | | | | | | |
| 101 | How old are you?(probe for best estimate) | ---------years | |  | | | |  |
| 102 | Age of the child | ----month | |  | | | |  |
| 103 | Sex of the child | 1. Male | | 2- Female | | | |  |
| 104 | Place of delivery | ------ | |  | | | |  |
| 105 | Birth weight | ------kg | |  | | | |  |
| 106 | What is your religion? | 1.Orthodox  2.Protestant | | 3.Muslim  4.catholic | | | | 5.others.specify ------ |
| 107 | Which ethnic group do you belong to? | 1.Amhara  3.Tigrie | | 2. Agew  4.Oromo | | | | 5.others.Specify------ |
| 108 | What is the highest education level you completed? | 1.Can not read  2.Can read &write | | 3.Primary  4.Secondary | | | | 5.College & above |
| 109 | What is your current occupation? | 1.House wife  2.Daily laborer | | 3.Farmer  4.G/ employee | | | | 5.Merchant  6.Other.specify |
| 110 | How many is your family size (Total number of family members)? | In number ------ | |  | | | |  |
| 111 | What is your current marital status? | 1.Married  2.Single | | 3.Widowed  4.Divorsed | | | | 5.Other,specify--- |
| 112 | What is the highest education level your husband completed? | 1.Can not read  2.Can read &write | | 3.Primary  4.Secondary | | | | 5.College & above |
| 113 | What is your husband’s current occupation? | 1.P/employee  2.Daily laborer | | 3.Farmer  4.G/ employee | | | | 5.Merchant  6.Other(specify) |
| 114 | Who is head of the household? | 1.Husband only | | 2.Woman only | | | | 3. Both |
| Table 2:Household Variables | | | | | | | | |
| **S.No** | **Variable** | | **Response** | | | | | |
| 201 | What type of house does this household have? | | 1.Corrugated iron sheet house  2.Grass roof house | | | | | |
| 202 | Do you have separate room for sleeping? | | 1. Yes | | 2. No |  | | |
| 203 | If yes for Q202, how many rooms? | | 1.One | | 2.Two | 3.Three | | |
| 204 | Do you have a separate room for animals? | | 1. Yes | | 2. No |  | | |
| 205 | Do you have a separate room which is used as a kitchen? | | 1. Yes | | 2. No |  | | |
| 206 | Does any member of this household own cows? | | 1. Yes | | 2. No | If yes, how many? | | |
| 207 | Does any member of this household own oxen? | | 1. Yes | | 2. No | If yes, how many? | | |
| 208 | Does any member of this household own calves? | | 1. Yes | | 2. No | If yes, how many? | | |
| 209 | Does any member of this household own other cattle? | | 1. Yes | | 2. No | If yes, how many? | | |
| 210 | Does any member of this household own horses? | | 1. Yes | | 2. No | If yes, how many? | | |
| 211 | Does any member of this household own donkeys? | | 1. Yes | | 2. No | If yes, how many? | | |
| 212 | Does any member of this household own mules? | | 1. Yes | | 2. No | If yes, how many? | | |
| 213 | Does any member of this household own sheep? | | 1. Yes | | 2. No | If yes, how many? | | |
| 214 | Does any member of this household own goats? | | 1. Yes | | 2. No | If yes, how many? | | |
| 215 | Does any member of this household own chickens? | | 1. Yes | | 2. No | If yes, how many? | | |
| 216 | Does any member of this household own beehives? | | 1. Yes | | 2. No | If yes, how many? | | |
| 217 | Does any member of this household own any agricultural land? | | 1. Yes | | 2. No | If yes, how many hectare? | | |
| 218 | Does any member of this household own Radio? | | 1. Yes | | 2. No | If yes, how many? | | |
| 219 | Does any member of this household own Television? | | 1. Yes | | 2. No | If yes, how many? | | |
| 220 | Does any member of this household own Telephone (Home)? | | 1. Yes | | 2. No | If yes, how many? | | |
| 221 | Does any member of this household own Telephone (Mobile)? | | 1. Yes | | 2. No | If yes, how many? | | |
| 222 | Does any member of this household own table? | | 1. Yes | | 2. No | If yes, how many? | | |
| 223 | Does any member of this household own chair? | | 1. Yes | | 2. No | If yes, how many? | | |
| 224 | Does any member of this household own bed with cotton/sponge/spring mattress? | | 1. Yes | | 2. No | If yes, how many? | | |
| 225 | Does any member of this household own a kerosene lamp/pressure lamp? | | 1. Yes | | 2. No | If yes, how many? | | |
| 226 | Does any member of this household own watch? | | 1. Yes | | 2. No | If yes, how many? | | |
| 227 | Does any member of this household own bicycle? | | 1. Yes | | 2. No | If yes, how many? | | |
| 228 | Does any member of this household own motorcycle or motor scooter? | | 1. Yes | | 2. No | If yes, how many? | | |
| 229 | Does any member of this household own animal-drawn cart? | | 1. Yes | | 2. No | If yes, how many? | | |
| 230 | Does any member of this household own car/ truck? | | 1. Yes | | 2. No | If yes, how many? | | |
| 231 | Does any member of this household own boat with a motor? | | 1. Yes | | 2. No | If yes, how many? | | |
| 232 | Does any member of this household own Bajaj? | | 1. Yes | | 2. No | If yes, how many? | | |
| 233 | Does any member of this household have a bank account? | | 1. Yes | | 2. No | If yes, how many? | | |
| 234 | Does any member of this household own khat farm? | | 1.Yes 2.No | | If yes, how many hectare? | | | |
| 235 | Does any member of this household own eucalyptus farm? | | 1.Yes 2.No | | If yes, how many hectare? | | | |
| 236 | How many quintals of the following cereals did the family produced in this year? | | 1.Teff--  2.Millet-  3.Maize- | | 4.Wheat—  5.Barelly--  6.Rye - | | 7.Rice  8.Other specify | |
| 237 | How many quintals of the following legume did the family produced in this year? | | 1.Bean--  2.Pea-- | | 3. Lentil-  4. Nut - | | 5. Chick pea--  6. Grass pea-  7.Other specify-- | |
| 238 | How many kilograms of vegetables did the family produced? | | 1.Gomen  2.Carrot  3. Potato | | 4. Tomato  5. Cabbage  6. Onion  10. Garlic | | 7.Beat root  8. Pumpkin  9.Pepper  11.Other specify | |
| 239 | How many kilograms of fruits did the family produced? | | 1.Mango 2.Banana  3.Avocado | | 4. Papaya  5. Guava  6.Lemon | | 7. Other specify- | |

Table 3: Obstetric and medical history

| S.No | **Variable** | **Response** | |
| --- | --- | --- | --- |
| 301 | How many times have you been pregnant so far? | In number ------ |  |
| 302 | How many times did you give birth so far? | In number ------- |  |
| 303 | Did you have plan to the current pregnancy? | 1.Yes | 2.No |
| 304 | Did you have ANC visit to the current pregnancy? | 1.Yes | 2.No |
| 305 | If yes for Q304, when did you start ANC visit? | --------weeks |  |
| 306 | If yes for Q304, how many visits do you have so far? | ------- |  |
| 307 | Do you have nausea during pregnancy? | 1. Yes, how sever is it? | 2.No |
| 308 | Do you have vomiting during pregnancy? | 1. Yes, how sever is it? | 2.No |
| 309 | Do you have any illness in the past two weeks? | 1.Yes | 2.No |
| 310 | If yes for Q314. Which symptom did/do you have? |  |  |
| 311 | If yes for Q315. What measure did you take to manage it? | --------- |  |
| 312 | Do you take coffee? | 1.Yes | 2.No |
| 313 | If yes for Q318, how Often do you drink coffee? | --------/day | ----/week |
| 314 | If yes for Q318, how many cups? | --------/day | ----/week |
| 315 | Did/do you take Iron/folic acid tablet? | 1.Yes | 2.No |
| 316 | If yes for Q320, when you started to take? | ------weeks |  |
| 317 | If yes for Q320, how often? | ---day, -----/week | ---/month |
| 318 | If yes for Q320, how long did you take it? | ------------ |  |
| 319 | For woman who did not take iron/folic acid tablet daily, what could be the reason for refraining from taking it? | ------------------- |  |
| 320 | Have you got counseling on maternal nutrition during this pregnancy? | 1.Yes | 2.No |
| 321 | If yes for Q325, who provide nutrition counseling? | 1.HP 2. HEWs | 3.Other |
| 322 | Distance of the nearby health institution from your home is? | ----- |  |

Table 4: infant and young child feeding practice

| **S.No** | **Variable** | **Response** | |
| --- | --- | --- | --- |
| 401 | Do you ever breast feed? | 1.Yes | 2. No |
| 402 | If yes to Q401, when do you start? | ------ |  |
| 403 | How long do you exclusively breast feed? | ----- |  |
| 404 | How long do you breast feed? | ---------------- |  |
| 405 | How many times do you breast feed /24hours? | ---- |  |
| 406 | When do you start complementary feeding? | ---------------- |  |
| 407 | How many times do you give complementary feeding for 6-8 month baby/24 hours? | ------- |  |
| 408 | How many times do you give complementary feeding for 9-12 month baby/24 hours? |  |  |
| 409 | How many times do you give complementary feeding for 12-24 month baby/24 hours? |  |  |
| 410 | How do you serve complementary food? |  |  |
| 411 | Do you give prelacteal feeding? |  |  |

Table 5: Environmental factors

| **S.No** | **Variable** | **Response** | | | |
| --- | --- | --- | --- | --- | --- |
| 501 | What is the main source of drinking water for members of your household? | 1. Piped water  2. Protected well water  3. Unprotected well water  4. River  5. Lake | | 6. Protected spring  7. Unprotected spring  8. Pond  9.Stream  10. Other, specify | |
| 502 | What is the main source of water used by your household for other purposes such as cooking and hand washing? | 1. Piped water  2. Protected well water  3. Unprotected well water  4. River  5. Lake | | 6. Protected spring  7. Unprotected spring  8. Pond  9. Other, specify | |
| 503 | Do you use any means to treat drinking water? | 1. Yes 2.No | | | |
| 504 | If yes for Q703, what do you usually use to make the water safer to drink? | 1. Boiling 2. Add chlorine 3. Use water filter | | | |
| 505 | Does your household have toilet? | 1.Yes 2.No | | | |
| 506 | Does your household use the toilet? |  | | | |
| 507 | If yes for Q705, what kind of toilet does your household have? | 1. Pit latrine with slab, private  2. Pit latrine without slab private  3. Pit latrine with slab, shard | | | 4. Pit latrine without slab, shared  5. No latrine |
| 508 | Is there hand washing facility in the toilet? | 1. Yes, which material?------- 2. No | | | |
| 509 | DO you wash Your hand after toilet? | 1.Yes 2. No | | | |
| 510 | Which material do you use to wash your hands after toilet? | 1. Water only  2. Soap with water sometimes | 3. Soap with water always 4.Ash with water | | |
| 511 | When do you washed your hand? | ---------- |  | | |
| 512 | How do you disposal HHs waste? | 1. Open field disposal  2. In a pit | 3.Burning | | |

Table 6: Household Food Insecurity Access Scale (HFIAS) Measurement Tool

| **S.No** | **Variable** | **Response** |
| --- | --- | --- |
| **601** | In the past four weeks, did you worry that your household would not have enough food? | 1. Yes 2. no |
|  | How often did this happen? | 1 = Rarely (once or twice in the past four weeks)  2 = Sometimes (3-10 times in the past four weeks)  3 = Often (>10times in the past four weeks) |
| **602** | In the past four weeks, were you or any household member not able to eat the kinds of foods you preferred because of a lack of resources? | 1. Yes 2. No |
|  | How often did this happen? | 1 = Rarely (once or twice in the past four weeks)  2 = Sometimes (3-10 times in the past four weeks)  3 = Often (>10times in the past four weeks) |
| **603** | In the past four weeks, did you or any household member have to eat a limited variety of foods due to a lack of resources? | 1. Yes 2. No |
|  | How often did this happen? | 1 = Rarely (once or twice in the past four weeks)  2 = Sometimes (3-10 times in the past four weeks)  3 = Often (>10times in the past four weeks) |
| **604** | In the past four weeks, did you or any household member have to eat some foods that you really did not want to eat because of lack of resource to obtain other types of food? | 1. Yes 2. No |
|  | How often did this happen? | 1 = Rarely (once or twice in the past four weeks)  2 = Sometimes (3-10 times in the past four weeks)  3 = Often (>10times in the past four weeks) |
| **605** | In the past four weeks, did you or any household member have to eat a smaller meal than you felt you needed because there was not enough food? | 1. Yes 2. No |
|  | How often did this happen? | 1 = Rarely (once or twice in the past four weeks)  2 = Sometimes (3-10 times in the past four weeks)  3 = Often (>10times in the past four weeks) |
| **606** | In the past four weeks, did you or any other household member have to eat fewer meals in a day because there was not enough food? | 1. Yes 2. No |
|  | How often did this happen? | 1 = Rarely (once or twice in the past four weeks)  2 = Sometimes (3-10 times in the past four weeks)  3 = Often (>10times in the past four weeks) |
| **607** | In the past four weeks, was there ever no food to eat of any kind in your household because of lack of resources to get food? | 1. Yes 2. No |
|  | How often did this happen? | 1 = Rarely (once or twice in the past four weeks)  2 = Sometimes (3-10 times in the past four weeks)  3 = Often (>10times in the past four weeks) |
| **608** | In the past four weeks, did you or any household member go to sleep at night hungry because there was not enough food? | 1. Yes 2. No |
|  | How often did this happen? | 1 = Rarely (once or twice in the past four weeks)  2 = Sometimes (3-10 times in the past four weeks)  3 = Often (>10times in the past four weeks) |
| **609** | In the past four weeks, did you or any household member go a whole day and night without eating anything because there was not enough food? | 1. Yes 2. No |
|  | How often did this happen? | 1 = Rarely (once or twice in the past four weeks)  2 = Sometimes (3-10 times in the past four weeks)  3 = Often (>10times in the past four weeks) |

Table 7: Measurements

| S.No | Variable | Response |
| --- | --- | --- |
| 701 | Weight of the child | -----kg |
| 702 | Height/length of the child | -----cm |
| 703 | Illness in the last 2 weeks? | 1.Yes 2. no |
| 704 | Sign and symptom |  |
| 705 | Measures taken |  |
